# Successful vaginal delivery in a parturient with long QT syndrome type 2 using double-catheter epidural analgesia: A CARE-compliant case report

**DOI:** 10.1097/MD.0000000000027790

**Published:** 2021-11-24

**Authors:** Hironori Ishizaki, Hiroaki Murata, Takuji Maekawa, Taiga Ichinomiya, Tetsuya Hara

**Affiliations:** a Department of Anesthesiology and Intensive Care Medicine, Nagasaki University Graduate School of Biomedical Sciences, 1-7-1 Sakamoto, Nagasaki, Japan; b Department of Anesthesiology, Nagasaki University Hospital, 1-7-1 Sakamoto, Nagasaki, Japan.

**Keywords:** epidural labor analgesia, landiolol, long QT syndrome type 2, vaginal delivery

## Abstract

**Rationale:**

Congenital long QT syndrome (LQTS) can cause syncope or sudden death due to ventricular arrhythmia. Congenital LQTS has 3 major types, 1, 2, and 3. Life-threatening arrhythmias are triggered by emotion in patients with LQTS type 2. As patients with LQTS type 2 have a higher incidence of postnatal cardiac events, careful perinatal management especially during delivery is required. To the best of our knowledge, perinatal management of a patient with LQTS type 2 has not been properly described with consideration to its type-specific risk factors for ventricular tachyarrhythmia.

**Patient concerns:**

A 36-year-old pregnant woman, gravida 1, para 0, with LQTS type 2 was scheduled to undergo vaginal delivery under epidural labor analgesia in the 38th week of pregnancy. No fainting episodes were reported since she began to take 40 mg of propranolol once daily at the age of 25. Despite this, we instituted maximum preventive measures for the safety of both the parturient and the fetus to minimize the risk of maternal cardiac events throughout the perinatal period.

**Diagnoses:**

She was diagnosed with LQTS type 2 by genetic testing at the age of 25.

**Interventions:**

Two epidural catheters were placed at levels T11–T12 and L5–S1. Injection of 0.2% ropivacaine and subsequent infusion of ropivacaine 0.1% with fentanyl (2 μg/mL) was directed through each catheter according to the stage of labor. Concurrently, landiolol, a selective and short-acting β1 receptor antagonist, was infused intravenously at a dose of 1 to 7 μg/kg/min.

**Outcomes:**

The delivery proceeded uneventfully without pain. No adverse cardiac events were observed during the perinatal period.

**Lessons:**

Vaginal delivery under epidural labor analgesia using 2 catheters might be a viable option for maternal perinatal care and delivery of patients with LQTS type 2.

## 1 Introduction

The prevalence of congenital long QT syndrome (LQTS) is approximately 1:2000.^[1,2]^ Congenital LQTS is caused by a mutation in the genes that encode cardiac ion channels^[3]^ and is clinically characterized by a prolonged QT interval on electrocardiogram and polymorphic ventricular tachyarrhythmia.^[4]^ Although most episodes of ventricular tachyarrhythmia are transient, few lead to fatal arrhythmias, such as torsades de pointes (Tdp), resulting in sudden death.^[4,5]^ Congenital LQTS types 1, 2, and 3 (LQT1, LQT2, and LQT3, respectively) account for approximately 80% of all gene mutation-related LQTS.^[6]^ Life-threatening arrhythmias are triggered by exercise in LQT1; emotion and auditory stimuli in LQT2^[7]^; and sleep/rest in LQT3 patients.^[3]^ β-blockers are the first choice to control the QT interval and prevent Tdp in patients with congenital LQTS.^[8]^

Although labor-associated strong emotional and physical stress is considered a risk factor for fatal arrhythmia in pregnant women with LQTS, the appropriate management of delivery has not yet been established. Moreover, no report has described the perinatal management of LQTS patients with focus on gene mutation type-specific risk stratification. We report the case of a pregnant woman with LQT2 who underwent painless vaginal delivery with double-catheter epidural labor analgesia and a concurrent intravenous infusion of landiolol, a selective and short-acting β1 receptor antagonist, to prevent ventricular arrhythmia.

## 2 Case report

The patient provided written consent, and the Ethics Committee of Nagasaki University Hospital approved this study (approval number 16122626). A 36-year-old pregnant woman (height, 158 cm; weight, 60 kg), gravida 1, para 0, with LQT2 had a history of repeated fainting on several occasions, triggered by diverse stimuli, such as hearing an alarm. At the age of 25, the QT interval corrected by the heart rate (QTc) was 500 ms, and she was diagnosed with LQT2 by genetic testing. She began to take 40 mg of propranolol once daily with good adherence. Subsequently, she did not experience any fainting episodes. Scheduled vaginal delivery under epidural labor analgesia was planned in the 38th week of pregnancy. Results of the physical examination and laboratory investigations, including hematology, biochemistry, chest radiography, and echocardiography, were normal. The electrocardiogram was normal except for a prolonged QTc (524 ms). The fetus was normal on examination. The parturient continued taking her regular dose of propranolol until the morning of delivery.

Every time the patient was taken into a procedure room, a calm environment was ensured by restricting the unnecessary entrance of medical staff. Special care was taken to keep her away from sudden and loud noises, such as ringing cell phones, monitor alarms, and the sound from the intercom broadcasts. Moreover, we talked to her in a tranquil tone throughout the procedure. An emergency cart and defibrillator were at the ready in every procedure room.

On the day prior to scheduled delivery, we placed 2 epidural catheters at T11–12 and L5–S1 in the operating room. One milliliter of 2% lidocaine was administered in each catheter to confirm proper placement of the epidural catheter and spread of local anesthetic. Loss of sensation from T8 to T10 and L5 to S1 was confirmed 15 minutes after each injection.

During delivery in the operating room, we evaluated the arterial pressure and arterial pressure-based cardiac output using the FloTrac/Vigileo system (Edwards Lifesciences, Irvine, CA) in addition to standard monitoring. Arterial blood samples were regularly analyzed for electrolytes. The fetal heart rate was measured with an intrapartum fetal monitor. Three milliliters of 0.2% ropivacaine was injected through an epidural catheter placed at T11–T12 upon induction of labor with oxytocin. Fifteen minutes later, hypesthesia from T10 to L1 was confirmed. Subsequently, continuous infusion of 0.1% ropivacaine with 2 μg/mL fentanyl was initiated at 6 mL/h, and the infusion rate was adjusted between 2 and 6 mL/h according to the analgesic area and labor intensity while communicating with the patient and obstetrician. As labor progressed, 6 mL of 0.2% ropivacaine was injected, and 0.1% ropivacaine with 2 μg/mL fentanyl (6–8 mL/h) was infused through the epidural catheter placed at L5–S1 before the full dilatation of the cervix. Epidural ropivacaine infusion through the catheters was discontinued following delivery of the placenta, and the catheters were removed. Continuous intravenous infusion of landiolol was started at a rate of 1 μg/kg/min upon induction of labor, and the infusion rate was titrated between 1 and 7 μg/kg/min at the discretion of the anesthesiologists.

Continuous intravenous infusion of oxytocin was initiated at a low dose (0.12 U/h) and then gradually increased to a maximum of 2.5 U/h with strict monitoring of the hemodynamic states of the patient and the fetus. Lithotomy position was adopted upon full cervical dilatation. Instrumental labor was applied to reduce maternal stress. The delivery proceeded uneventfully without pain, except for abdominal strain, and without anesthetic or obstetric complications. The elapsed time from the initiation of labor induction to the full dilatation of the cervix and from the full dilatation of the cervix to completion was 6 and 1 hour, respectively. During the entire delivery period, there were no significant changes in QTc, blood pressure, heart rate, cardiac index, or blood electrolyte levels. The heart rate of the baby immediately after birth was 160 beats per minute. Apgar scores were 9 and 10 at 1 and 5 minutes following delivery, respectively. The estimated blood loss, including amniotic fluid, was 500 mL, the urine output was 1550 mL, and the total fluid volume of intravenous infusion was 2390 mL during delivery. No postpartum analgesics were required. Landiolol infusion was maintained following delivery at a rate of 3 μg/kg/min until the patient resumed oral propranolol. The post-parturition clinical course was uneventful, and no ventricular arrhythmia was observed throughout the perinatal period. Overall, the patient was completely satisfied with the management of vaginal delivery with epidural analgesia using double epidural catheter and the perinatal management to avoid cardiac events related to LQT2 with careful attention and adequate medication.

## 3 Discussion

In patients with LQT2, female sex, age 15 to 40 years, and QTc >500 ms despite β-receptor blockade are risk factors of cardiac events.^[9–11]^ The present patient presented with all these. Patients with LQT2 are more likely to have cardiac events than LQT1 patients despite use of β-blockers.^[10]^ A history of cardiac events is an independent risk factor for subsequent syncope or probable LQTS-related death before the age of 50 years.^[12]^ Moreover, the incidence of postnatal heart accidents in LQT2 patients reaches approximately 4- and 10-fold compared with that in LQT1 and LQT3 patients, respectively.^[13]^ Considering these, the perinatal management of the present patient required strict attention to avoid life-threatening cardiac events. However, only one report on a parturient with LQTS has mentioned the specific genotype (LQT1) of the patient.^[14]^ In another report, a woman was suspected of having LQT1 considering the occurrence of a syncopal episode.^[15]^ Early planning for the management of delivery in patients with congenital LQTS, considering genotype-specific characteristics and perinatal risk for cardiac events is paramount to decrease the likelihood of catastrophic outcomes.^[3,13]^

Although caesarean section is preferred for delivery in parturient with LQTS,^[14–16]^ postoperative pain is one of the biggest concerns for postnatal emotional stress and is difficult to manage compared with vaginal delivery. Thus, we opted for vaginal delivery with epidural analgesia in this patient. Although the combined spinal-epidural technique with an epidural catheter at L2–L3 was a satisfactory analgesic option, a higher infusion rate was required to obtain adequate analgesia.^[17]^ Consequently, we deemed it reasonable to use the double epidural catheter technique^[18]^ to obtain reliable and controllable labor analgesia with less amount of local anesthetic. Epidural local anesthetic infusion through the upper catheter at T11–T12 mainly addresses visceral pain from cervical dilation, while infusion through the lower catheter at L5–S1 mainly addresses somatic pain associated with dilation of the birth canal. Delivery under epidural analgesia can cause uterine inertia or prolonged delivery, resulting in conversion to caesarean section. To address this concern without sacrificing the quality of analgesia, we planned to reduce or discontinue the local anesthetic infusion via the upper catheter, independent of the use of the lower catheter, when uterine inertia was suspected.

Landiolol, a selective and short-acting β1 receptor antagonist, has been reported to be useful for heart rate control during birth because it had no inhibitory effects on uterine contraction through β2 receptor blockade.^[15]^ The administration of β-blockers had been effective in preventing ventricular arrhythmia in the present patient, and patients with LQT2 have a high incidence of postnatal heart accidents^[13]^; thus, we prophylactically administered landiolol from induction of labor until oral propranolol could be resumed following delivery. No adverse events related to the use of landiolol were observed.

Oxytocin is a drug that induces uterine contraction and prevents bleeding during delivery. Bolus injection of oxytocin is known to cause QTc prolongation; hence, oxytocin is considered a potentially arrhythmogenic agent among patients at risk of Tdp. Slow administration of oxytocin has been recommended.^[16,19]^ Herein, oxytocin infusion was initiated at a low rate and gradually titrated, resulting in appropriate uterine contraction without QTc prolongation.

As a limitation of this case report, we did not directly show the preventive effect of each intervention. For example, single epidural catheter might be enough to provide sufficient analgesia during vaginal delivery. Likewise, only regular dose of propranolol until the morning of delivery might be effective during delivery without intravenous landiolol. We provided the maximum preventive measures for the safety of both the parturient and the fetus to minimize the risk of maternal cardiac events throughout the perinatal period.

## 4 Conclusion

We achieved painless vaginal delivery for a parturient with LQT2 using double-catheter epidural analgesia. Moreover, careful infusion of oxytocin and landiolol was useful in avoiding perinatal ventricular arrhythmias.

## Acknowledgments

The authors would like to thank Editage (www.editage.com) for English language editing.

## Author contributions

**Conceptualization:** Hironori Ishizaki, Hiroaki Murata.

**Data curation:** Hironori Ishizaki, Takuji Maekawa.

**Supervision:** Hiroaki Murata, Tetsuya Hara.

**Writing – original draft:** Hironori Ishizaki, Takuji Maekawa.

**Writing – review & editing:** Hiroaki Murata, Taiga Ichinomiya, Tetsuya Hara.
